# Liquid-liquid phase separation in super enhancer-driven oncogenesis: mechanisms, immune evasion, and therapeutic implications

**DOI:** 10.3389/fcell.2025.1645325

**Published:** 2025-11-14

**Authors:** Ruoxi Yu, Deyu Sun, Chuang Zhang

**Affiliations:** Department of Radiotherapy Oncology, Cancer Hospital of Dalian University of Technology, Liaoning Cancer Hospital and Institute, Cancer Hospital of China Medical University, Shenyang, China

**Keywords:** liquid-liquid phase separation (LLPS), super-enhancers (SEs), transcriptional regulation, cancer Therapy, immune evasion

## Abstract

Liquid-Liquid Phase Separation (LLPS) allows biomolecules to form membrane-less organelles or condensates through weak multivalent interactions. Recent studies have revealed the critical role of LLPS in super-enhancer (SE)-associated tumorigenesis, progression, and immune evasion. This mini-review summarizes recent advances in the role of LLPS in SE-driven oncogenic and immune evasion processes, while discussing its potential therapeutic implications in cancer. Current evidence indicates that LLPS regulates the expression of tumor-associated genes through multiple mechanisms, such as facilitating transcription factor recruitment, promoting chromatin looping, modulating chromatin topology, and maintaining chromatin accessibility. Notably, LLPS-associated SEs functionally regulate not only tumor cells but also immune cells within the tumor microenvironment, contributing to tumor immune evasion. Thus, understanding the relationship between LLPS and SEs is crucial for elucidating the mechanisms underlying tumor initiation and progression. These insights may provide foundational evidence for developing novel anti-tumor therapeutic agents.

## 1 Introduction

Super-enhancers (SEs), as higher-order chromatin regulatory elements, have garnered significant attention in recent years. They are large complexes composed of multiple adjacent enhancers that recruit high concentrations of transcription factors (TFs) and coactivators to form dense transcriptional regulatory condensates. Through this, SEs efficiently regulate the expression of critical genes, particularly those involved in cell identity determination and disease pathogenesis (Chapuy et al., 2013; Di Micco et al., 2014).

Liquid-liquid phase separation (LLPS) is a process by which biomolecules (e.g., proteins and nucleic acids) form membrane-less organelles or condensates through weak multivalent interactions, including hydrogen bonds, hydrophobic forces, and electrostatic interactions—a concept established by foundational studies on biomolecular condensates (Banani et al., 2017; Banani et al., 2016). A key driver of LLPS, particularly in proteins, is the presence of intrinsically disordered regions (IDRs)—flexible domains lacking a fixed three-dimensional (3D) structure that facilitate the dynamic, multivalent contacts necessary for phase separation. LLPS enhances the efficiency of molecular interactions and regulates physiological functions such as gene expression, signal transduction, and material transport (Sabari et al., 2018).

Biomolecular condensates formed through LLPS exhibit high molecular density, dynamic behavior, and compartmentalization—properties that align with the transcriptional regulatory features of SEs. The role of LLPS in organizing diverse cellular processes, and its dysregulation in cancer pathogenesis, have been extensively reviewed (Tong et al., 2022). Consequently, a growing number of studies are now focusing on the role of LLPS in SE-driven oncogenic processes (Table 1). For instance, Sabari et al. (2018) first revealed the central role of phase separation in the functional regulation of SEs. They found that transcriptional coactivators Bromodomain-containing protein 4 (BRD4) and Mediator Complex Subunit 1(MED1) undergo LLPS to form dynamic condensates at SE regions. These condensates spatially compartmentalize the transcriptional machinery (e.g., RNA polymerase II, mediator complex) to maintain sustained expression of cell identity-related genes, such as pluripotency genes in stem cells. This groundbreaking discovery opened a new chapter in the study of LLPS in SEs, highlighting its critical contribution in SE assembly, coordinated gene activation, and disease-associated dysregulation.

**TABLE 1:** Comparison of shared features between liquid-liquid phase separation (LLPS) and super-enhancers (SEs).

Similarity	LLPS	SEs
High molecular density	LLPS forms high-density molecular condensates or foci, such as RNA–protein condensates	SEs are characterized by the dense enrichment of transcription factors, co-factors, and diverse gene regulatory molecules within specific chromatin regions
Compartmentalization	LLPS creates membrane-less compartments via physical segregation, thereby enhancing the local concentration of relevant molecules to facilitate efficient biochemical reactions	SEs compartmentalize numerous gene transcription regulators into distinct chromatin domains, which promotes transcription initiation and activation
Dynamic nature	As a dynamic physicochemical process, LLPS allows condensates to rapidly assemble and disassemble, with continuous updating and exchange of their components	The molecular assembly of SEs exhibits high dynamicity, involving the continuous recruitment and release of factors to regulate the spatiotemporal specificity of gene expression
Complex regulatory mechanisms	LLPS is finely regulated by various factors, including molecular concentration, temperature, ionic strength, and weak interactions (e.g., electrostatic, hydrophobic, and van der waals forces)	SEs are governed by a complex regulatory network comprising multiple transcription factors, coactivators, and epigenetic enzyme modifications
Sensitivity to cellular state	LLPS is influenced by diverse cellular conditions, such as pH, ionic strength, molecular concentration, and ATP levels, as well as cellular signaling pathways	SEs are modulated by cellular metabolic cues (e.g., acetyl-CoA levels), epigenetic modifications (e.g., H3K27ac), and cellular signaling cascades
Multivalent interaction mechanisms	LLPS relies on weak interactions between multivalent molecules, such as proteins containing intrinsically disordered regions and RNA.	SEs require the multimerization of transcription factors (e.g., BRD4 dimers) and collaborative binding with chromatin structures (e.g., CTCF-mediated chromatin looping)
Disease association	Aberrant LLPS has been implicated in the pathogenesis of neurodegenerative diseases, cancers, and other pathological conditions	SE dysregulation is closely associated with the development of cancers, autoimmune diseases, and other pathological states

This mini-review summarizes current progress in understanding the critical involvement of LLPS in SE-mediated tumor progression and immune evasion, and discusses its potential applications in emerging cancer therapies. The keywords “super-enhancer” and “phase-separation” were used to search the PubMed database, which yielded approximately 40 relevant publications. Following this initial automated search, a manual screening process was performed to identify studies closely related to both LLPS and tumor biology. The selected literature was then systematically reviewed to synthesize the current understanding of SE composition, regulation, and oncogenic functions.

## 2 Mechanisms of LLPS in regulating SE-Mediated transcriptional activation

**FIGURE 1:**
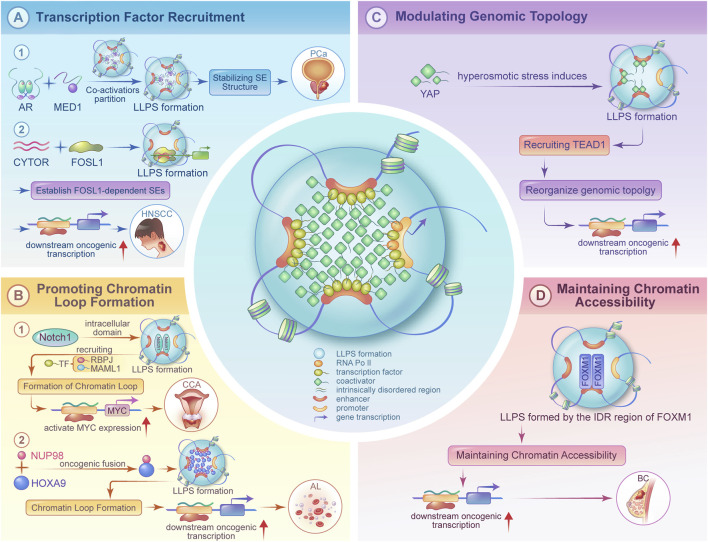
Schematic representation illustrating the mechanisms of liquid–liquid phase separation (LLPS) in super-enhancer (SE)-mediated transcriptional activation. Key abbreviations: AR (Androgen Receptor), MED1 (Mediator Complex Subunit 1), CYTOR (Cytoskeleton Regulator RNA), FOSL1 (FOS Like 1, AP-1 Transcription Factor Subunit), HNSCC (Head and Neck Squamous Cell Carcinoma), RBPJ (Recombination Signal Binding Protein For Immunoglobulin Kappa J Region), NUP98 (Nucleoporin 98), HOXA9 (Homeobox A9), YAP (Yes1 Associated Transcriptional Regulator), TEAD1 (TEA Domain Transcription Factor 1), FOXM1 (Forkhead Box M1), IDR (Intrinsically Disordered Region).

Accumulating literature has demonstrated that LLPS regulates gene expression, exhibiting diverse mechanisms that drive SE functionality. Its operational modes primarily encompass the following four categories (Figure 1):

1. Facilitating TF Recruitment: LLPS increases local molecular concentrations at SE regions by forming condensates, thereby promoting efficient TF binding to DNA sites. For example, in prostate cancer, androgen receptor forms phase-separated condensates under androgen stimulation, enriching TFs at SEs to drive tumor progression (Zhang et al., 2023a). Similarly, in head and neck squamous cell carcinoma, the long noncoding RNA (lncRNA) CYTOR stabilizes FOS Like 1 (FOSL1)-dependent transcriptional condensates at SEs (FOSL1 is an AP-1 TF subunit), promoting SE-driven expression of metastasis-related genes (e.g., *MMP9*) (Wang et al., 2024) (Figure 1A).
2. Promoting Chromatin Looping: LLPS-driven condensates can function as architectural hubs that direct the formation of specific, long-range chromatin loops. This point-to-point connection brings distal regulatory elements (such as enhancers) into spatial proximity with their target gene promoters, thereby facilitating precise transcriptional activation. A key example is found in acute myeloid leukemia, where the NUP98-HOXA9 fusion protein undergoes LLPS to drive the assembly of chromatin loops at oncogene-associated SEs (e.g., near *MEIS1*), bypassing the canonical loop formation mechanism mediated by CCCTC-Binding Factor (Ahn et al., 2021) (Figure 1B).
3. Modulating Chromatin Topology: Beyond facilitating specific loops, LLPS can orchestrate large-scale, global reorganization of the 3D chromatin architecture. This includes remodeling the topological domains and creating specialized nuclear compartments conducive to high-level transcription. For instance, the Hippo pathway effector YAP forms nuclear condensates that collectively reshape the 3D genome to enhance chromatin interactions and sustain the expression of target genes (e.g., *CTGF*, *CYR61*) (Cai et al., 2019). Furthermore, nucleoporin NUP153 anchors SEs near nuclear pore complexes through LLPS, effectively creating a distinct transcription-prone nuclear sub-compartment that amplifies the expression of oncogenes such as *TP63* (Hazawa et al., 2024). The Notch1 intracellular domain (NICD) also forms transcriptional condensates via phase separation-coupled permeation to activate Notch target genes (Foran et al., 2024) (Figure 1C).
4. Maintaining Chromatin Accessibility: LLPS enriches chromatin remodeling factors to maintain an open state at SE regions. In breast cancer, FOXM1 forms condensates through IDR1-mediated phase separation, recruiting histone acetyltransferases (e.g., p300) and chromatin helicases to enhance SE accessibility and promote the expression of tumor stemness genes (e.g., *SOX2*). (Xie et al., 2025). Interestingly, the functional conservation of LLPS has also been confirmed in virology: respiratory syncytial virus forms phase-separated replication factories known as inclusion bodies. Small-molecule drugs that can “solidify” these inclusion bodies inhibit viral replication, offering potential cross-species parallels for targeting LLPS in cancer therapies (Risso-Ballester et al., 2021) (Figure 1D).

However, current research on LLPS mechanisms in SEs remains preliminary. A prevailing hypothesis is that LLPS forms dynamic condensates at SEs, increasing local molecular concentrations and compartmentalizing TFs. This hypothesis is strongly supported by a recent genomic-scale analysis, which demonstrated that TFs with high intrinsic phase separation ability preferentially exhibit clustered genomic binding patterns at cell-specific SEs. This provides a fundamental principle for the formation of transcriptional condensates (Wang et al., 2025). Seminal work has demonstrated that SE-associated LLPS condensates selectively enrich key transcriptional components (e.g., Mediator, RNA Polymerase II) while excluding others, thereby amplifying transcriptional efficiency (Sabari et al., 2018). However, direct evidence for LLPS in maintaining chromatin accessibility is limited and indirect. Anchoring SEs near nuclear pores to enhance mRNA export efficiency provides a spatiotemporal explanation for LLPS functionality. Overall, deeper mechanistic insights and direct evidence are needed to elucidate the role of LLPS in SE regulation.

## 3 Key structural domains involved in LLPS

The core of LLPS-driven SE functionality lies in multivalent interactions mediated by specific protein domains. These domains determine phase separation thresholds, condensate properties (e.g., fluidity, permeability), and integrate microenvironmental signals (e.g., metabolites, post-translational modifications) to dynamically regulate SE activity. Based on current literature, the primary structural/functional domains can be categorized into the following three types:

1. IDRs: IDRs are protein segments lacking a stable, fixed 3D structure, which confers high flexibility and enables dynamic, multivalent interactions—key physicochemical features that drive LLPS. Consequently, IDRs are recognized as one of the most critical domains facilitating biomolecular condensation. For example, the IDRs of the NUP98-HOXA9 fusion protein form condensates that induce chromatin looping and activate oncogene expression (Ahn et al., 2021). The IDR of Anillin Actin Binding Protein interacts with RNA polymerase II and promotes transcriptional condensate formation to regulate gene expression (Cao et al., 2025). Thus, the key role of IDRs in LLPS formation has been widely recognized, and IDR content has gradually become an important indicator for determining phase-separation potential.
2. Zinc Finger Domain 3 (ZF3): In addition to IDRs, zinc finger domains have also been confirmed as key regions that promote LLPS. In lung adenocarcinoma, the C-terminal ZF3 domain of Specificity Protein 1 is essential for its phase-separation ability and promotes tumor progression by modulating the transcription of Regulator of G protein Signaling 20 gene (Shan et al., 2024). However, current research on the role of zinc finger domains in phase separation is still in its early stages, and further investigation is needed to understand their specific regulatory mechanisms and synergistic effects with other domains.
3. RNA Recognition Motif 1 (RRM1): In neuroblastoma, the RRM1 domain of Non-POU Domain-Containing Octamer-Binding Protein (NONO) is critical for LLPS and RNA binding, determining the cellular localization and function of the protein. NONO mutants lacking RRM1 cannot bind to RNA and form larger, spherical condensates. This indicates that RRM1 significantly affects both phase-separation and gene regulatory functions by modulating RNA-binding ability (Zhang et al., 2023b).

Although IDRs remain the most well-established drivers of LLPS, protein domains such as RRM1 and ZF3 have also been confirmed to promote this process. From a physicochemical perspective, these domains have dynamic interaction interfaces that enable weak interactions, providing a foundation for LLPS. Moreover, they can integrate microenvironmental signals to dynamically regulate the transcriptional activity of SEs. Researchers speculate that this property may stem from their common physicochemical characteristics, such as charge distribution or hydrogen-bond donors and acceptors, which facilitate multivalent interactions and thereby influence phase-separation behavior. However, these hypotheses require further exploration and verification.

## 4 Key components influencing LLPS in SEs

**FIGURE 2:**
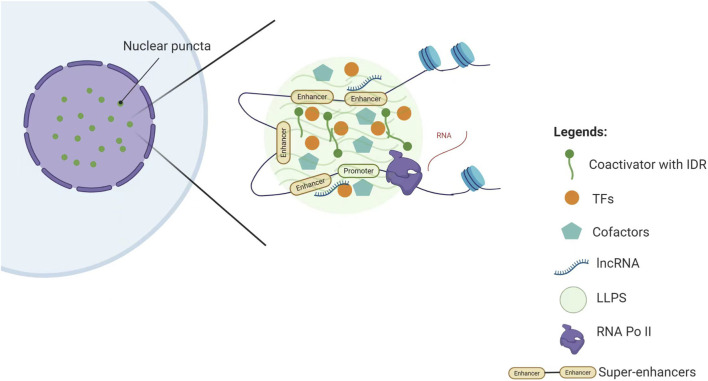
Schematic representation of the components in SE-associated transcriptional complexes that promote LLPS. Key abbreviations: LLPS (Liquid-Liquid Phase Separation), TFs (Transcription Factors), IDR (Intrinsically Disordered Region) Note: This figure was created using BioRender.com.

With continued research, several components in SE-associated transcriptional complexes have been identified as LLPS promoters, thus driving tumor progression (Figure 2).

1. Condensation Hub Function of Coactivators: As core coactivators in SE regulation, MED1 and BRD4 mediate LLPS through their IDRs, forming dynamic transcriptional condensates. These condensates spatially compartmentalize and enrich the transcriptional machinery, thereby promoting the sustained high expression of pluripotency genes such as *OCT4* and *NANOG* (Sabari et al., 2018).
2. Dynamic Regulation Network of TFs: In addition to coactivators, TFs can form specialized SE regulatory nodes through LLPS. For instance, FOXM1 maintains chromatin accessibility through IDR-mediated phase separation, activating pro-metastatic SE-related genes (e.g., *MMP9*, *VEGFA*) and promoting aggressive breast cancer progression (Xie et al., 2025). In osteosarcoma, HOXB8 and FOSL1 form transcriptional condensates that restructure the 3D topology of SEs, abnormally activating chemoresistance-related genes (e.g., *ABCG2*, *BCL2*) (Lu et al., 2021). These findings indicate that TFs can influence SE function through LLPS.
3. Synergistic Amplification Effect of Cofactors: Cofactors act as “signaling amplifiers” for SE activity through phase separation. In hepatocellular carcinoma, the cofactor p300 forms condensates with Twist1 and YY1 at the *miR-9* SE, creating a local high-concentration interaction hub that continuously activates *miR-9* expression drives malignant progression (Meng et al., 2023). This suggests that cofactors enhance the basal activity of SEs and also promote tumor progression through LLPS.
4. Topological Organizing Role of lncRNA: Certain lncRNA function as “invisible architects” in the SE regulation network, participating in LLPS through unique molecular interaction modes. For example, the lncRNA CYTOR interacts with the TF FOSL1 to promote the formation of FOSL1-based phase-separated condensates, which in turn establish FOSL1-dependent SEs. This process promotes tumor budding, stemness, and metastasis in head and neck squamous cell carcinoma (Wang et al., 2024), suggesting that lncRNA may be a key component in maintaining LLPS stability.

Despite these findings, research on the components of SE-associated transcriptional complexes promoting LLPS remains limited, with most studies focusing on specific molecular functions. Existing studies indicate several components that regulate SE function via LLPS, suggesting a more universal role for this process in SE formation. As research deepens and techniques improve, more such components are expected to be identified.

Notably, LLPS may not only involve specific TFs or cofactors, but also many other potential SE-related molecules. However, broader conclusions cannot yet be drawn, as current research is still in its early stages, harboring several limitations. With the application of high-throughput techniques, substantial evidence can be uncovered to provide strong support for the hypothesis, clarifying the central role and widespread function of LLPS in SE regulation. This path of research may offer new insights into gene expression regulation and provide important clues for disease treatment-related research.

## 5 Targeting LLPS to inhibit SE function

Recent studies have confirmed that SEs are critically involved in tumor proliferation, survival, invasion, and immune evasion (Berico et al., 2023; Chen et al., 2024; Zhao et al., 2024; Shi et al., 2025; Wei et al., 2024; Shi et al., 2023; Hazan et al., 2019). Strategies for treating tumors by inhibiting SE function have made significant progress in recent years, with a central focus on interfering with the dynamic process of LLPS. Current research mainly revolves around three directions: (1) directly disrupting SE transcriptional condensates, (2) regulating the phase separation capabilities of key proteins, and (3) developing combination therapies to overcome drug resistance and delivery bottlenecks.

For example, Cyclin-Dependent Kinase (CDK) inhibitors such as THZ1 target CDK7/12/13 to disrupt SE condensates and suppress oncogenic genes (e.g., *Brachyury*). However, their clinical efficacy is limited by the physical barriers of LLPS condensates—hydrophobic cores and dynamic permeability lead to low drug enrichment efficiency. Similar limitations apply to Bromodomain and Extra-Terminal motif (BET) inhibitors (e.g., JQ1) (Sheppard et al., 2021; Li et al., 2023; Zhang et al., 2024).

To address this delivery challenge, innovative solutions have been proposed in the field of bioengineering. These strategies aim to overcome the very properties of LLPS that confer its function—spatiotemporal compartmentalization and high local concentration. For example, the targeted nanoparticle system for FOXM1 condensates in breast cancer regulates molecular hydrophobicity to break through the physical barriers and achieve a therapeutically effective local drug concentration within the condensates (Xie et al., 2025). Another strategy focuses on regulating the phase separation capabilities of key proteins. For instance, in hepatocellular carcinoma, metformin activates Adenosine Monophosphate-activated Protein Kinase (AMPK) to induce Twist1 phosphorylation. This disrupts its LLPS-mediated interactions with YY1 and p300, thereby suppressing SE-driven *miR-9* expression and successfully reversing malignant phenotypes (Meng et al., 2023).

The complexity of targeting this axis is further highlighted by emerging resistance mechanisms. For instance, SE-driven Nuclear Receptor Subfamily 3 Group C Member 1 (NR3C1) forms phase-separated condensates that promote 5-fluorouracil (5-FU) resistance in gastric cancer. Combined inhibition of SEs and NR3C1 improves 5-FU efficacy, providing direct pre-clinical support for multi-target therapeutic strategies (Yu et al., 2025). Additionally, the lncRNA HANR colocalizes with PIP2 in the perinucleolar compartment, a hallmark of cancer cell nuclei. This localization correlates with oncogenic SE markers, implicating lncRNA–lipid condensate components as potential alternative factors in oncogenic nuclear organization and adaptive resistance (Miladinović et al., 2024).

Combination therapy strategies have emerged to address the limitations of monotherapy. In castration-resistant prostate cancer, lysine-specific demethylase 1 (LSD1) inhibitors reduce chromatin accessibility to disrupt SE structure. When combined with BET inhibitors, they synergistically inhibit androgen receptor signaling and delay drug resistance (Li et al., 2023; Zhang et al., 2024). A more direct example is the combination of JQ1 and 1,6-hexanediol, where JQ1 blocks the binding of BET proteins to acetylated histones and 1,6-hexanediol disrupts the hydrophobic interactions that stabilize the fusion protein condensates of promyelocytic leukemia/retinoic acid receptor alpha. Their combined use has been shown to increase the complete remission rate in acute promyelocytic leukemia (Zhang et al., 2024).

Although such strategies are effective, they also face challenges such as additive toxicity (e.g., bone marrow suppression) and insufficient universality across tumor types. Future breakthroughs may depend on spatiotemporally precise delivery, such as the development of stimulus-responsive nanoparticles (e.g., ROS/pH-sensitive carriers). These delivery systems release drugs specifically in the tumor microenvironment, thereby reducing off-target effects (Xie et al., 2025).

## 6 LLPS in SE-Driven radiation and chemotherapy resistance

LLPS has been implicated in cancer cell resistance to radiation. Radiation-induced DNA damage can trigger the formation of condensates that isolate and protect key repair proteins, thereby enhancing cell survival. For example, Matsumoto et al. (2024) revealed that SEs formed through LLPS of BRD4 confer intrinsic radio-resistance to the genome. BRD4 degraders, such as ARV-771, disrupt these condensates and significantly sensitize cancer cells to radiation, providing a novel strategy for radiotherapy (Matsumoto et al., 2024).

Additionally, LLPS is involved in chemotherapy resistance related to SEs. For instance, in gastric cancer, researchers analyzed the SE landscape of 5-FU-resistant and 5-FU-sensitive cells and found that NR3C1 is the main TF driving SEs, with its high expression associated with 5-FU resistance. Inhibiting NR3C1 or disrupting SEs restores the sensitivity of gastric cancer cells to 5-FU, underscoring the key contribution of LLPS in regulating SE-related chemoresistance (Yu et al., 2025). Similarly, a recent study in small cell lung cancer established FOXP1-mediated LLPS as a central chemoresistance mechanism. FOXP1 forms phase-separated condensates that regulate the SP8 SE, activating the homologous recombination repair pathway to promote drug resistance. This phenomenon directly links transcriptional condensates to treatment failure (Tang et al., 2025).

However, whether radiation or chemotherapy directly induces phase separation is debated. Some scholars believe that DNA damage triggers stress-signaling pathways such as Ataxia-Telangiectasia Mutated/ATM and Rad3-Related to indirectly regulate LLPS. Therefore, future research must combine single-cell sequencing and live-cell imaging to parse dynamic response mechanisms.

## 7 LLPS in SE-Driven immune evasion

Recent studies have begun to link LLPS, SEs, and tumor immunology. Anti-tumor immunity involves complex interactions among various cells and molecules, requiring precise regulation of gene expression. As an intracellular fine-tuning mechanism, LLPS can dynamically regulate transcriptional activity and enhancer states through phase separation, highlighting its potential importance in this field.

For instance, in breast cancer, FOXM1 undergoes LLPS to bind Forkhead consensus DNA elements. This process recruits transcriptional machinery, maintains chromatin accessibility and the SE landscape, and promotes tumor metastasis and growth. However, AMPK-mediated FOXM1 phosphorylation can disrupt its condensed state, reducing oncogenic transcriptional activity and activating innate immune responses, thereby enhancing tumor immunogenicity (Xie et al., 2025). In macrophages, Zinc Finger MYND-Type Containing 8 (ZMYND8) and NF-κB/p65 form liquid condensates upon lipopolysaccharide stimulation. Acetylation of p65 further enhances condensate formation, guiding ZMYND8 redistribution to inflammation-related SEs and recruiting LSD1 to suppress their activity. This process is crucial for precisely regulating macrophage-mediated immune responses (Jia et al., 2021).

Collectively, these findings indicate that LLPS finely regulates gene expression through interactions with SEs, thereby influencing the tumor immune microenvironment. Future studies must explore the functional differences of LLPS across various immune and tumor cells to develop precise therapeutic strategies targeting LLPS.

## 8 Discussion

LLPS has been recognized as a key regulator of SE-driven transcriptional programs in cancer. Its ability to stabilize SEs, modulate chromatin structure, and promote transcriptional activation highlights its importance in tumor development. The synergistic action of LLPS and SEs also provides new insights into the mechanisms of tumor immune evasion.

However, current understanding remains fragmented owing to several critical knowledge gaps, raising relevant questions:

1. Model Systems: Which experimental models (e.g., patient-derived organoids or *in vivo* imaging models) are most suited for dissecting the causal role of LLPS in SE-driven oncogenesis within a pathophysiological context?
2. Methodological Limitations: How can we overcome current technical barriers, such as distinguishing functional condensates from non-specific aggregates in fixed cells, and achieving real-time, high-resolution visualization of LLPS dynamics in living cells?
3. Targeting Specificity: What are the definitive molecular features that create cancer-specific vulnerabilities in transcriptional condensates, and how can we exploit them to design small-molecule inhibitors that selectively disrupt oncogenic LLPS without affecting global transcription?

Addressing these questions will require the integration of advanced techniques, such as single-molecule imaging and optogenetic controls, with interdisciplinary collaboration to translate LLPS biology into novel therapeutic paradigms.

## Glossary

SEs: super-enhancers
TF: transcription factor
LLPS: liquid-liquid phase separation
BRD4: Bromodomain-containing protein four
MED1: Mediator Complex Subunit 1
AR: androgen receptor
lncRNA: long noncoding RNA
FOSL1: FOS Like 1, AP-1 transcription factor subunit
CTCF: CCCTC-Binding Factor
NPCs: nuclear pore complexes
NICD: Notch1 intracellular domain
PSCP: phase separation-coupled permeation
IDR1: first intrinsically disordered region
RSV: Respiratory syncytial virus
IBs: inclusion bodies
IDRs: Intrinsically Disordered Regions
ANLN: Anillin Actin Binding Protein
Pol II: RNA polymerase II
ZF3: Zinc Finger Domain three
SP1: Specificity Protein 1 (SP1)
RGS20: Regulator of G protein Signaling 20
RRM1: RNA Recognition Motif 1
NONO: Non-POU Domain-Containing Octamer-Binding Protein
HCC: hepatocellular carcinoma
HNSCC: Head and Neck Squamous Cell Carcinoma
CDK: Cyclin-Dependent Kinase
BET: Bromodomain and Extra-Terminal motif family
AMPK: Adenosine Monophosphate-activated Protein Kinase
LSD1: lysine-specific demethylase 1
PML/RARα: promyelocytic leukemia/retinoic acid receptor alpha
5-FU: 5-fluorouracil
NR3C1: Nuclear Receptor Subfamily 3 Group C Member 1
ATM/ATR: ataxia-telangiectasia mutated/ATM and Rad3-related
FKH: Forkhead
ZMYND8: Zinc Finger MYND-Type Containing eight
LPS: lipopolysaccharide

## References

[B1] Ahn J. H. Davis E. S. Daugird T. A. Zhao S. Quiroga I. Y. Uryu H. (2021). Phase separation drives aberrant chromatin looping and cancer development. Nature 595, 591–595. 10.1038/s41586-021-03662-5 34163069 PMC8647409

[B2] Banani S. F. Rice A. M. Peeples W. B. Lin Y. Jain S. Parker R. (2016). Compositional control of phase-separated cellular bodies. Cell 166, 651–663. 10.1016/j.cell.2016.06.010 27374333 PMC4967043

[B3] Banani S. F. Lee H. O. Hyman A. A. Rosen M. K. (2017). Biomolecular condensates: organizers of cellular biochemistry. Nat. Rev. Mol. Cell Biol. 18, 285–298. 10.1038/nrm.2017.7 28225081 PMC7434221

[B4] Berico P. Nogaret M. Cigrang M. Lallement A. Vand-Rajabpour F. Flores-Yanke A. (2023). Super-enhancer-driven expression of BAHCC1 promotes melanoma cell proliferation and genome stability. Cell Rep. 42, 113363. 10.1016/j.celrep.2023.113363 37924516

[B5] Cai D. Feliciano D. Dong P. Flores E. Gruebele M. Porat-Shliom N. (2019). Phase separation of YAP reorganizes genome topology for long-term YAP target gene expression. Nat. Cell Biol. 21, 1578–1589. 10.1038/s41556-019-0433-z 31792379 PMC8259329

[B6] Cao Y. F. Wang H. Sun Y. Tong B. B. Shi W. Q. Peng L. (2025). Nuclear ANLN regulates transcription initiation related pol II clustering and target gene expression. Nat. Commun. 16, 1271. 10.1038/s41467-025-56645-9 39894879 PMC11788435

[B7] Chapuy B. Mckeown M. R. Lin C. Y. Monti S. Roemer M. G. Qi J. (2013). Discovery and characterization of super-enhancer-associated dependencies in diffuse large B cell lymphoma. Cancer Cell 24, 777–790. 10.1016/j.ccr.2013.11.003 24332044 PMC4018722

[B8] Chen Y. Zhuo R. Sun L. Tao Y. Li G. Zhu F. (2024). Super-enhancer-driven IRF2BP2 enhances ALK activity and promotes neuroblastoma cell proliferation. Neuro Oncol. 26, 1878–1894. 10.1093/neuonc/noae109 38864832 PMC11449008

[B9] DI Micco R. Fontanals-Cirera B. Low V. Ntziachristos P. Yuen S. K. Lovell C. D. (2014). Control of embryonic stem cell identity by BRD4-dependent transcriptional elongation of super-enhancer-associated pluripotency genes. Cell Rep. 9, 234–247. 10.1016/j.celrep.2014.08.055 25263550 PMC4317728

[B10] Foran G. Hallam R. D. Megaly M. Turgambayeva A. Antfolk D. Li Y. (2024). Notch1 phase separation Coupled percolation facilitates target gene expression and enhancer looping. Sci. Rep. 14, 21912. 10.1038/s41598-024-71634-6 39300145 PMC11413390

[B11] Hazan I. Monin J. Bouwman B. A. M. Crosetto N. Aqeilan R. I. (2019). Activation of oncogenic super-enhancers is coupled with DNA repair by RAD51. Cell Rep. 29, 560–572. 10.1016/j.celrep.2019.09.001 31618627 PMC6899447

[B12] Hazawa M. Ikliptikawati D. K. Iwashima Y. Lin D. C. Jiang Y. Qiu Y. (2024). Super-enhancer trapping by the nuclear pore *via* intrinsically disordered regions of proteins in squamous cell carcinoma cells. Cell Chem. Biol. 31, 792–804.e7. 10.1016/j.chembiol.2023.10.005 37924814

[B13] Jia P. Li X. Wang X. Yao L. Xu Y. Hu Y. (2021). ZMYND8 mediated liquid condensates spatiotemporally decommission the latent super-enhancers during macrophage polarization. Nat. Commun. 12, 6535. 10.1038/s41467-021-26864-x 34764296 PMC8586003

[B14] Li M. Liu M. Han W. Wang Z. Han D. Patalano S. (2023). LSD1 inhibition disrupts super-enhancer-driven oncogenic transcriptional programs in castration-resistant prostate cancer. Cancer Res. 83, 1684–1698. 10.1158/0008-5472.CAN-22-2433 36877164 PMC10192194

[B15] Lu B. Zou C. Yang M. He Y. He J. Zhang C. (2021). Pharmacological inhibition of core regulatory circuitry liquid-liquid phase separation suppresses metastasis and chemoresistance in osteosarcoma. Adv. Sci. (Weinh) 8, e2101895. 10.1002/advs.202101895 34432948 PMC8529446

[B16] Matsumoto K. Ikliptikawati D. K. Makiyama K. Mochizuki K. Tobita M. Kobayashi I. (2024). Phase-separated super-enhancers confer an innate radioresistance on genomic DNA. J. Radiat. Res. 65, 482–490. 10.1093/jrr/rrae044 38874522 PMC11262858

[B17] Meng J. Han J. Wang X. Wu T. Zhang H. An H. (2023). Twist1-YY1-p300 complex promotes the malignant progression of HCC through activation of miR-9 by forming phase-separated condensates at super-enhancers and relieved by metformin. Pharmacol. Res. 188, 106661. 10.1016/j.phrs.2023.106661 36669583

[B18] Miladinović A. Antiga L. Venit T. Bayona-Hernandez A. Červenka J. Labala R. K. (2024). The perinucleolar compartment and the oncogenic super-enhancers are part of the same phase-separated structure filled with phosphatidylinositol 4,5bisphosphate and long noncoding RNA HANR. Adv. Biol. Regul. 95, 101069. 10.1016/j.jbior.2024.101069 39648081

[B19] Risso-Ballester J. Galloux M. Cao J. LE Goffic R. Hontonnou F. Jobart-Malfait A. (2021). A condensate-hardening drug blocks RSV replication *in vivo* . Nature 595, 596–599. 10.1038/s41586-021-03703-z 34234347

[B20] Sabari B. R. Dall'Agnese A. Boija A. Klein I. A. Coffey E. L. Shrinivas K. (2018). Coactivator condensation at super-enhancers links phase separation and gene control. Science 361, eaar3958. 10.1126/science.aar3958 29930091 PMC6092193

[B21] Shan L. Wang W. DU L. Li D. Wang Y. Xie Y. (2024). SP1 undergoes phase separation and activates RGS20 expression through super-enhancers to promote lung adenocarcinoma progression. Proc. Natl. Acad. Sci. U. S. A. 121, e2401834121. 10.1073/pnas.2401834121 38976739 PMC11260144

[B22] Sheppard H. E. Dall'Agnese A. Park W. D. Shamim M. H. Dubrulle J. Johnson H. L. (2021). Targeted brachyury degradation disrupts a highly specific autoregulatory program controlling chordoma cell identity. Cell Rep. Med. 2, 100188. 10.1016/j.xcrm.2020.100188 33521702 PMC7817874

[B23] Shi C. Chen L. Pi H. Cui H. Fan C. Tan F. (2023). Identifying a locus in super-enhancer and its resident NFE2L1/MAFG as transcriptional factors that drive PD-L1 expression and immune evasion. Oncogenesis 12, 56. 10.1038/s41389-023-00500-3 37985752 PMC10662283

[B24] Shi Z. Wang R. Huang J. Qian Q. Hu M. Zhang H. (2025). Super-enhancer-driven ameboidal-type cell migration-related MMP14 expression in tongue squamous cell carcinoma switched by BATF and ATF3. J. Pharm. Pharmacol. 77, 64–75. 10.1093/jpp/rgae063 38836550

[B25] Tang Y. Niu Y. Chen Y. Zhou X. Hu Y. Sun L. (2025). Targeting FOXP1 phase separation in small cell lung cancer mechanisms of chemotherapy resistance. Commun. Biol. 8, 431. 10.1038/s42003-025-07804-7 40082538 PMC11906602

[B26] Tong X. Tang R. Xu J. Wang W. Zhao Y. Yu X. (2022). Liquid-liquid phase separation in tumor biology. Signal Transduct. Target Ther. 7, 221. 10.1038/s41392-022-01076-x 35803926 PMC9270353

[B27] Wang W. Yun B. Hoyle R. G. Ma Z. Zaman S. U. Xiong G. (2024). CYTOR facilitates formation of FOSL1 phase separation and super enhancers to drive metastasis of tumor budding cells in head and neck squamous cell carcinoma. Adv. Sci. (Weinh) 11, e2305002. 10.1002/advs.202305002 38032139 PMC10811474

[B28] Wang S. Wang Z. Zang C. (2025). Genomic clustering tendency of transcription factors reflects phase-separated transcriptional condensates at super-enhancers. Nucleic Acids Res. 53, gkaf015. 10.1093/nar/gkaf015 39868536 PMC11760973

[B29] Wei X. Liu J. Cheng J. Cai W. Xie W. Wang K. (2024). Super-enhancer-driven ZFP36L1 promotes PD-L1 expression in infiltrative gastric cancer. Elife 13. 10.7554/eLife.96445 39373630 PMC11458174

[B30] Xie F. Zhou X. Ran Y. Li R. Zou J. Wan S. (2025). Targeting FOXM1 condensates reduces breast tumour growth and metastasis. Nature 638, 1112–1121. 10.1038/s41586-024-08421-w 39814884

[B31] Yu J. Chen M. Sang Q. Li F. Xu Z. Yu B. (2025). Super-enhancer activates master transcription factor NR3C1 expression and promotes 5-FU resistance in gastric cancer. Adv. Sci. (Weinh) 12, e2409050. 10.1002/advs.202409050 39731339 PMC11831572

[B32] Zhang F. Biswas M. Massah S. Lee J. Lingadahalli S. Wong S. (2023a). Dynamic phase separation of the androgen receptor and its coactivators key to regulate gene expression. Nucleic Acids Res. 51, 99–116. 10.1093/nar/gkac1158 36535377 PMC9841400

[B33] Zhang S. Cooper J. A. Chong Y. S. Naveed A. Mayoh C. Jayatilleke N. (2023b). NONO enhances mRNA processing of super-enhancer-associated GATA2 and HAND2 genes in neuroblastoma. EMBO Rep. 24, e54977. 10.15252/embr.202254977 36416237 PMC9900351

[B34] Zhang Y. Lou J. Liu Y. Jin P. Tan Y. Song H. (2024). Phase separation of PML/RARα and BRD4 coassembled microspeckles governs transcriptional dysregulation in acute promyelocytic leukemia. Proc. Natl. Acad. Sci. U. S. A. 121, e2406519121. 10.1073/pnas.2406519121 39136995 PMC11348160

[B35] Zhao Y. Zhou Y. Qian Y. Wei W. Lin X. Mao S. (2024). m(6)A-dependent upregulation of DDX21 by super-enhancer-driven IGF2BP2 and IGF2BP3 facilitates progression of acute myeloid leukaemia. Clin. Transl. Med. 14, e1628. 10.1002/ctm2.1628 38572589 PMC10993053

